# Karyopherin abnormalities in neurodegenerative proteinopathies

**DOI:** 10.1093/brain/awab201

**Published:** 2021-05-21

**Authors:** Terouz Pasha, Anna Zatorska, Daulet Sharipov, Boris Rogelj, Tibor Hortobágyi, Frank Hirth

**Affiliations:** 1 King's College London, Institute of Psychiatry, Psychology and Neuroscience, Maurice Wohl Clinical Neuroscience Institute, Department of Basic and Clinical Neuroscience, Institute of Psychiatry, Psychology and Neuroscience, London SE5 9RT, UK; 2 Jozef Stefan Institute, Department of Biotechnology, University of Ljubljana, Faculty of Chemistry and Chemical Technology, 1000 Ljubljana, Slovenia; 3 Biomedical Research Institute BRIS, University of Ljubljana, Faculty of Chemistry and Chemical Technology, 1000 Ljubljana, Slovenia; 4 ELKH-DE Cerebrovascular and Neurodegenerative Research Group, Department of Neurology, University of Debrecen, 4032 Debrecen, Hungary; 5 King's College London, Department of Old Age Psychiatry, Institute of Psychiatry, Psychology and Neuroscience, London SE5 8AF, UK

**Keywords:** karyopherin, nucleocytoplasmic transport, phase transition, protein aggregation, neurodegeneration

## Abstract

Neurodegenerative proteinopathies are characterised by progressive cell loss that is preceded by the mislocalisation and aberrant accumulation of proteins prone to aggregation. Despite their different physiological functions, disease-related proteins like tau, alpha-synuclein, Tar DNA binding protein-43, Fused in sarcoma and mutant Huntingtin, all share low complexity regions that can mediate their liquid-liquid phase transitions. The proteins’ phase transitions can range from native monomers to soluble oligomers, liquid droplets and further to irreversible, often-mislocalised aggregates that characterise the stages and severity of neurodegenerative diseases. Recent advances into the underlying pathogenic mechanisms have associated mislocalisation and aberrant accumulation of disease-related proteins with defective nucleocytoplasmic transport and its mediators called karyopherins. These studies identify karyopherin abnormalities in amyotrophic lateral sclerosis, frontotemporal dementia, Alzheimer’s disease, and synucleinopathies including Parkinson’s disease and dementia with Lewy bodies, that range from altered expression levels to the subcellular mislocalisation and aggregation of karyopherin alpha and beta proteins. The reported findings reveal that in addition to their classical function in nuclear import and export, karyopherins can also act as chaperones by shielding aggregation-prone proteins against misfolding, accumulation and irreversible phase-transition into insoluble aggregates. Karyopherin abnormalities can, therefore, be both the cause and consequence of protein mislocalisation and aggregate formation in degenerative proteinopathies. The resulting vicious feedback cycle of karyopherin pathology and proteinopathy identifies karyopherin abnormalities as a common denominator of onset and progression of neurodegenerative disease. Pharmacological targeting of karyopherins, already in clinical trials as therapeutic intervention targeting cancers such as glioblastoma and viral infections like COVID-19, may therefore represent a promising new avenue for disease-modifying treatments in neurodegenerative proteinopathies.

**Author affiliations:**

**Running title**: Karyopherin pathology in proteinopathies

**Abbreviations:** AD = Alzheimer’s Disease; ADAR = Adenosine Deaminase RNA Specific; ALS = Amyotrophic Lateral Sclerosis; ARM = Armadillo domain; Aβ = Amyloid-beta; CSE1L/CAS = Chromosome Segregation 1 Like/Cellular apoptosis susceptibility; DLB =

Dementia with Lewy Bodies; EWS = Ewing Sarcoma; FG = phenylalanine and glycine; FTD = Fronto-Temporal Dementia; FUS = Fused in sarcoma; HD = Huntington's Disease; HTT = Huntingtin; IBB = Importin beta binding; IPOA = importin alpha; KPNA = karyopherin-α; KPNB = karyopherin-β; LB = Lewy body: LCR = low-complexity region; LN = Lewy neurites; MGC = Mammalian Gene Collection; NES = nuclear export signal; NF-κB = nuclear factor-κB; NFT = neurofibrillary tangles; NLS = nuclear localisation signal; NPC = nuclear pore complex; NPI = Nucleoprotein interactor; NRF2 = Nuclear factor erythroid 2-related factor 2; NT17 = N-terminal 17-residue sequence; NUP = nucleoporin; PD = Parkinson's Disease; PDD = Parkinson's disease dementia; PRKCI = Protein Kinase C Iota; PY-NLS = Proline-Tyrosine NLS; QIP = Importin alpha Q; RAN = Ras-related nuclear; RBM8A = RNA Binding Motif Protein 8A; RCC1 = regulator of chromosome condensation 1; RCH = RAG cohort protein; RP = ribosomal protein; Ran-GAP = Ran GTPase activating protein; RanBP1 = Ran binding protein 1; SFSR1/2 = splicing factors SR1/2; SINE = selective inhibitors of nuclear export; SNAI1 = Snail Family Transcriptional Repressor; SRP19 = Signal Recognition Particle 19; SRP = signal recognition particle; STAT12 = Signal transducer and activator of transcription 2; STAT1 = Signal transducer and activator of transcription 1; Scyl1 = SCY1 like pseudokinase 1; TAF15 = TATA binding associated factor 15; TDP-43 = transactive response DNA binding protein of 43 kDa; Ubc = ubiquitin-conjugating enzyme; cNLS = classical nuclear localisation signal; eIF1A = eukaryotic translation initiation factor 1A; hnRNP = Heterogeneous nuclear ribonucleoproteins A1/A2/H/M; mHTT = mutant huntingtin; p50RhoGAP = p50 Rho GTPase-activating protein; poly-P = poly-Proline; α-syn = alpha-synuclein.

## Introduction

Neurodegeneration is the selective and progressive loss of neurons, eventually leading to cognitive and behavioural deficits for which currently no cure or effective treatments are available.[1] Neurodegenerative diseases are distinguished by clinical appearance and pathology, such as movement disorders like Parkinson's Disease (PD), Huntington's Disease (HD) and Amyotrophic Lateral Sclerosis (ALS), and dementias like Alzheimer's Disease (AD), Fronto-Temporal Dementia (FTD) and Dementia with Lewy Bodies (DLB). Despite their clinical heterogeneity, the majority of neurodegenerative diseases are characterised by proteinaceous inclusions that are often mis-localized and accumulate in the extracellular milieu or intracellular compartments of affected cells. The disease-related proteins are frequently posttranslationally modified (phosphorylated, ubiquitinated, acetylated and cleaved into fragments), which is often related to their transition from native soluble monomers into oligomers, protofibrils and the ultimate deposition of β-sheet fibrils and insoluble aggregates.[2] The quantity and distribution of these aggregates correlates with the stage and severity of the diseases,[3,4] and different proteins aggregate in different neurodegenerative diseases. For example, in AD proteins aggregate as amyloid plaques comprising Amyloid-beta (Aβ) fragments of aberrantly cleaved amyloid precursor protein; in PD and DLB as Lewy bodies comprising aberrant forms of alpha-synuclein (α-syn); in AD, FTD, PD and ALS as tangles comprising phosphorylated tau; and in ALS, FTD, AD and PD as insoluble aggregates containing TAR DNA binding protein 43 (TDP-43), with some TDP-43-negative cases of ALS and FTD as insoluble aggregates of fused in sarcoma (FUS); and in HD as mutant huntingtin with aberrant poly-glutamine extension.

The disease-related proteins differ significantly in function and, until recently, it remained elusive how such functionally unrelated proteins can lead to comparable pathogenic alterations and degenerative cell death. However, structure-function analyes revealed the majority of these proteins harbor unstructured, intrinsically disordered amino acid sequences called low-complexity regions (LCRs), that have recently been linked with unprecedented insights to pathogenic mechanisms underlying neurodegenerative diseases.[5-11] These studies showed that LCRs of disease-related proteins enable their aberrant phase transition into liquids and the subsequent mislocalisation and accumulation as fibrillary aggregates.[12-14] Further mechanistic insights revealed that nuclear transport receptors, termed karyopherins, and their abnormalities are associated with protein mislocalisation and aggregation.[15,16]

These findings provide evidence that in addition to nucleocytoplasmic transport, karyopherins can also function as chaperones to maintain their protein cargos in a soluble non-aggregated state, thereby reducing aberrant phase transition and protein accumulation.[17-20] For example, in the case of ALS and FTD, it has been shown that the underlying pathogenesis involves defective nucleocytoplasmic transport across the nuclear envelope,[21-24] mediated by karyopherins known to shuttle proteins across the nuclear membrane.[25] These findings suggest a dual role of karyopherins as a molecular switch between protein aggregation states and as a gatekeeper of protein localisation, deregulation of which can trigger onset and progression of disease. Here we review recent evidence and current understanding of karyopherin function, their role in onset and progression of disease, and discuss their potential as therapeutic targets in neurodegenerative proteinopathies.

## Karyopherin protein families

Karyopherins are classified into karyopherin-α (KPNA) and karyopherin-β (KPNB) families (**Figure 1**), with each member recognising its own set of cargo proteins or RNAs.[26] KPNA orthologues are grouped into subfamilies, α1, α2 and α3 (**Figure 1A; Table 1**) based on differences in primary amino acid sequences.[27] The human genome encodes seven KPNA orthologues with a common structure consisting of ten helical Armadillo (ARM) repeats that recognise the nuclear localisation signal (NLS) of their specific cargos; a short C-terminal region which functions as a specific binding site for the export factor chromosome segregation 1 like (CSE1L, also known as Cellular apoptosis susceptibility, CAS); and an N-terminal Importin beta binding (IBB) domain for KPNB1 binding.[27] Evidence from cancer research reveals these subfamilies have distinct cargo specificities. For instance, members of the α2 subfamily KPNA3 and KPNA4 show particular affinity for NF-κB and the nuclear regulator of chromosome condensation 1 (RCC1), whilst KPNA1 of the α3 subfamily specifically binds to signal transducer and activator of transcription 1 and 2 (STAT1, STAT2).[28] Furthermore, the N-terminal IBB domain of KPNAs (**Figure 1A**) also control the accessibility of the nuclear localisation signal binding site by mimicking an NLS structure and folding back to occupy the ARM repeats when KPNAs are not bound to their cargos.[29,30] Thus, only cargos with high affinity are bound to KPNAs (**Table 1**). Cargos with a relatively lower affinity bind to the ARM repeats of KPNAs upon formation of KPNA-KPNB1 heterodimer, which frees the NLS binding site.[30]

KPNB proteins (**Figure 1B** and **Table 2**) have an essential role in nucleocytoplasmic transport and as a secondary consequence downstream of their role in nuclear transport also affect the regulation of gene expression, the immune response and signal transduction,[31] as well as viral pathogenesis and tumorigenesis.[32,33] Members of the KPNB family share an importin N-terminal domain responsible for RanGTP/GDP binding that drives active nucleocytoplasmic transport (**Figure 2**). KPNBs are characterized by multiple tandem helical repeats termed HEAT repeats located in the C-terminal region, each composed of two antiparallel helices A and B arranged into ring-like structures.[34,35] The HEAT repeats are responsible for binding to KPNA and to cargos.[36] In humans, the KPNB family contains 18 members (**Table 2**), which can be divided into three groups based on their major role in nucleocytoplasmic transport as either exportins, importins or for bidirectional transport. For example, KPNBs such as exportin 4 (XPO4) or exportin 5 (XPO5) mediate nuclear export of cargos,[37-41] whereas KPNB1 or TNPO1 and TNPO2 (also called KPNB2 and KPNB2B, respectively) mediate nuclear import (see **Table 2**, also for synonyms and cargos). Despite significant differences in protein structure, localisation and function of the majority of disease-associated proteins, their nucleocytoplasmic shuttling is mediated by karyopherins.

## Karyopherins in nucleocytoplasmic transport

The nucleus of cells provides a protective environment through tightly regulated protein import and export.[42] While smaller proteins as well as ions, salts and nucleotides can enter the nucleus via passive diffusion,[34] proteins larger than 40-65kDa require binding to nuclear transport receptors to traverse the nuclear pore complex (NPC).[26] This process (**Figure 2**) is regulated by three key elements: the nuclear pore complex, nuclear transport factors of the karyopherin families and a Ras-related nuclear (RAN) protein gradient.[43] The nuclear pore complex consists of three rings: the nucleoplasmic ring, the middle inner ring and the cytoplasmic ring that form an aqueous channel spanning the nuclear envelope.[44] At the molecular level, it consists of an assembly of approximately 30 nucleoporins (NUPs) classified into six groups based on their localisation.[45] The nucleoporins are also grouped by their molecular function into those that form the NPC scaffold, the trans-membrane nucleoporins and the phenylalanine and glycine (FG) containing nucleoporins – where phase separation occurs. In the absence of stable nucleoporin complexes, the nucleus would be pore-free with a continuous nuclear envelope.[43] Ran, a small GTPase protein, drives the direction of cargo transport, depending on its bound nucleotide state. The nuclear regulator RCC1 functions as a guanine nucleotide exchange factor and maintains RanGTP levels by facilitating GDP-GTP exchange. In the cytoplasm, Ran GTPase activating protein (Ran-GAP) and Ran binding protein 1 (RanBP1) catalyse the hydrolysis of GTP to GDP. Cooperation of these two systems forms a Ran gradient, which guides directionality of nucleopcytoplasmic transport (**Figure 2**) and facilitates the rapid and selective cargo transport into and out of the nucleus.[26]

Karyopherins are central to nucleocytoplasmic trafficking as they recognise the nuclear localisation signal or nuclear export signal (NES) of cargo proteins.[31] In the 'classical' nuclear import pathway,[46] cargo proteins such as TDP-43 contain a 'classical' NLS (cNLS), which

KPNAs bind to mediate nuclear import. cNLSs are either monopartite or bipartite sequences. Whilst monopartite sequences contain a single cluster of basic amino acids (Lys/Arg) as exemplified by the SV40 large T antigen NLS 126-PKKKRRV-132,[47] bipartite sequences are made up of two clusters of basic residues separated by an unconserved linker region (e. g. nucleoplasmin NLS 155-KRPAATKKAGQAKKKK-170).[48]

During classical nuclear import **(Figure 2)**, the cargo-KPNA bipartite complex binds to KPNB1 creating a trimeric complex (cargo-KPNA-KPNB1) that interacts with the FG motifs of the nucleoporins in the central channel, and subsequently, driven by the RanGTP/GDP gradient, this complex translocates to the nucleus.[42,49] In the nucleus the trimeric complex starts to dissociate once RanGTP binds to KPNB1, with the physical separation of KPNB1 and KPNA. Subsequent dissociation of the KPNA-cargo complex is slow and can be catalysed by CSE1L/CAS that also mediates export of KPNA. The KPNA, RanGTP and CSE1L/CAS proteins are then recycled back to the cytoplasm **(Figure 2)**. In addition to the cNLS-dependent pathway, other import pathways involve non-classical NLSs, which unlike cNLSs bind directly to KPNBs without the need for interaction with KPNA.[34] One such example is the Proline-Tyrosine NLS (PY-NLS) motif of FUS, which is directly imported via TNPO1 and 2 (KPNB2 and KPNB2B).[7,50,51] Although FUS and TDP-43 have putative nuclear export signal sequences, it should be noted that TDP-43 and FUS could also be exported through the nucleus via passive diffusion.[52,53] Bioinformatics analysis indicates that the nuclear export signals in TDP-43 and FUS are non-functional and both proteins can be exported independent of the export receptors XPO1 and XPO5.[52]

## Karyopherin abnormalities in neurodegenerative diseases

Karyopherin abnormalities have been implicated in ALS and FTD with TDP pathology;[15-20,54] in Alzheimer's disease and FTD with Tau pathology;[55-60] in synucleinopathies;[61-66] and in cell and animal models of Huntington's disease[67-70], suggesting karyopherin-associated pathology as a common denominator in the aetiology of neurodegenerative proteinopathies. In the following sections we review how karyopherin abnormalities manifest in these neurodegenerative diseases which is summarised in **Table 3**.

### Karyopherin abnormalities in Amyotrophic Lateral Sclerosis and Frontotemporal Dementia with TDP pathology

ALS and FTD are part of a common disease spectrum with overlapping clinical, pathological and genetic features.[71,72] ALS is a debilitating and ultimately fatal disease, characterised by the progressive loss of upper and lower motor neurons which inevitably leads to degeneration of corticospinal tracts and muscle wasting.[73,74] In contrast, FTD is characterised by progressive neuronal atrophy which occurs in the frontal and temporal lobes with the resulting loss of cortical tissue leading to personality and behavioural changes, and/or a gradual impairment of language skills.[72,75] A common pathological feature found in both diseases are abnormally aggregated cytoplasmic inclusions of TDP-43[76,77] that characterise 97% of ALS cases and approximately 45% of patients with FTD[71] which are therefore termed FTD-TDP in contrast to FTD cases with tau pathology termed FTD-Tau.

Reduced protein levels of KPNA2 and CAS are observed in FTD-TDP frontal cortex[54] and reduced KPNB1 levels are found in the spinal cord of patients with ALS.[78] Immunohistochemical analysis of nucleoporins in ALS revealed an irregular, fractured nuclear pore complex,[79] possibly preventing the nuclear import of TDP-43. In addition, irregular, disrupted nuclear staining for NUP62 or KPNB1 has been reported in sporadic ALS with TDP-43 mislocalisation providing further evidence for disruption of the nuclear pore complex in TDP-43 pathologies.[80] Moreover, cell culture experiments revealed that KPNB1 and CAS knockdown dysregulates the classical nuclear import of TDP-43, leading to its cytoplasmic accumulation comparable to disease pathology.[54]

Corresponding phenotypes were also observed in the most common genetic form of ALS and FTD caused by an intronic hexanucleotide repeat expansion (GGGGCC) in the *C9ORF72* gene, termed C9ALS/FTD.[21,22,81-84] Several pathogenic mechanisms have been related to the C9ORF72 mutation, including haploinsufficiency[85,86] and homozygous loss of *C9ORF72* function,[87] as well as toxic gain of function of the repeat RNA,[83,88-90] and the accumulation of dipeptide repeat proteins (DPRs) that are translated from G4C2 mRNA templates through repeat-associated non-ATG translation (RAN translation).[89,91] However, the exact contribution of each of these mechanisms to onset and progression of disease in C9ALS/FTD is still inconclusive.[92] Functional studies in C9ALS/FTD cell and animal models identified defective nucleocytoplasmic transport and nuclear pore complex deficits as potential pathogenic mechanisms underlying neurodegeneration.[21-24,93,94] These findings are supported by rescue experiments in yeast and *Drosophila* models of C9ALS/FTD, which identified importins, exportins, Ran-GTP cycle regulators and nuclear pore components as modifiers of G4C2 RNA and DPR-mediated neurodegeneration.[21,23,24] Moreover, a recent study, using a permeabilised cell assay, showed that arginine-rich DPRs bind KPNB1, ultimately leading to dose- and length-dependent disruption of nuclear import.[95] Of note, this study also revealed that transport disruption was neither due to sequestration of NCT components, nor to blockade of the FG nucleoporin-mediated permeability barrier, but rather due to perturbed KPNB-related cargo-binding.[95]

Comparable nucleocytoplasmic transport and nuclear pore complex deficits were also reported in models of TDP-43 proteinopathy.[15,16] These studies provide experimental evidence that accumulating cytosolic TDP-43 causes cytoplasmic mislocalization of KPNAs in *Drosophila* models of C9ALS/FTD, a phenotype that is also observed in post-mortem frontal cortex tissue of C9FTD patients and sporadic cases of FTD-TDP.[15,16] Cytosolic TDP-43 functions in the stabilisation, translation and transport of mRNA.[96] However, imbalanced cytoplasmic accumulation of TDP-43 can induce its nuclear depletion and aggregate formation,[97] cellular phenotypes that are both related to onset and progression of disease.[98] Nuclear depletion of TDP-43, through decreased expression or aggregate formation, has also been shown to lead to reduced levels of RanBP1, suggesting that TDP-43 mislocalisation may be both cause and consequence of errors in nucleocytoplasmic transport.[98] However, the initiating causes leading to cytoplasmic accumulation of TDP-43 remain elusive.

Interaction studies have identified direct protein-protein interactions between TDP-43 and KPNA2[54] and KPNA4[15], in addition to TDP-43 targeting the pre-mRNA of KPNA4 and to a lesser extent that of KPNA2.[99] Furthermore, cytoplasmic TDP-43 droplets that are not associated with stress granules have been shown to sequester KPNA4 and NUP62 and induce mislocalization of RanGap1, Ran, and NUP107.[20] These findings support the feedback loop model proposed by Solomon *et al.*[16], whereby cytoplasmic, non-aggregated droplets of TDP-43 recruit karyopherins which in turn causes nucleocytoplasmic transport deficits. Of note, KPNAs also bind arginine-rich DPRs,[93] the most toxic DPR species observed in models of C9ALS/FTD,[100,101] which in turn impairs KPNA/KPNB mediated nuclear import, including cargo transport of TDP-43.[102] Moreover, cell culture studies revealed that DPRs can be sequestered into cytoplasmic inclusions by β-sheet protein aggregates, including fragments of

TDP-43.[70] Furthermore, experiments in cell culture[54] and in *Drosophila*[103] revealed the depletion of KPNB1 and KPNAs directly contributes to impaired nuclear import and cytoplasmic accumulation of TDP-43. These data suggest a vicious cycle of TDP-43 and karyopherin abnormalities[16] and in turn impaired nucleocytoplasmic transport and a defective nuclear pore complex, that trigger onset and progression of disease in ALS and FTD-TDP.

### Karyopherin abnormalities in Alzheimer's Disease and Frontotemporal Dementia with Tau pathology

Alzheimer's disease is the most common cause of dementia, accounting for up to 75% of all dementia cases.[104] Patients suffer from insidiously progressive disturbances in memory and other cognitive capabilities such as dysfunction in language, visuospatial deficits and impaired executive function.[105] AD-related proteinopathy is characterized by neurofibrillary tangles enriched in hyperphosphorylated tau, and amyloid plaques comprising Aβ fragments of aberrantly cleaved Amyloid precursor protein.[4,106] Karyopherin abnormalities in Alzheimer's disease have been repeatedly reported, yet how they contribute to disease formation and/or progression is only starting to emerge.

Accumulated and aggregated KPNA2/Importin-α1 has been identified in neurofibrillary tangles[57] and in Hirano bodies in hippocampal CA1 neurons of AD patient cases.[56] cDNA microarray studies using AD patient brain samples identified abnormally upregulated levels of KPNA3,[55] while KPNA6 upregulation was identified in association with small non-coding RNAs in AD-affected brain.[58] In addition to KPNA abnormalities, several members of the KPNB family have been found to be mis-localised, accumulated or aggregated in Alzheimer's disease patient brain. KPNB1 and KPNB2 were found in cytoplasmic granules in hippocampal neurons, while KPNB2 accumulation was also detected in tangle-bearing cells of Alzheimer's disease cases.[107] Expression of KPNB1 and XPO5/exportin-5 were both found to be upregulated in AD cases and both proteins co-localized with hyperphosphorylated tau.[59] These data suggest that karyopherin abnormalities may play a direct role in tau pathology related to Alzheimer's disease and possibly also FTD-Tau.

In support of this hypothesis, experiments using a *Drosophila* model of tauopathy revealed that tau-induced nuclear envelope invaginations sequestered the karyopherin beta family member XPO1/exportin-1.[108] Another study using AD patient brain material together with transgenic mouse models of FTD-Tau identified a direct link between tau pathology and nucleocytoplasmic transport deficits.[60] Using co-immunoprecipitation from human AD brain tissue, these studies demonstrated direct binding between tau and Nup98 and that tau interacts with nuclear pore proteins enriched in phenylalanine-glycine (FG) repeat domains. In addition, these FG-motif nucleoporins were shown to mis-localize from and in turn disrupt the nuclear membrane in primary cortical neurons derived from transgenic mouse models of FTD-Tau.[60] As a likely consequence of these NPC deficits, alterations in active protein import and export were observed including decreased expression of Ran required for energy-dependent nucleocytoplasmic shuttling.[60] This reduction in Ran expression and reduced nucleocytoplasmic transport has also been observed in an earlier study using Alzheimer's disease brain samples and neuroblastoma cells,[109] suggesting that nucleocytoplasmic transport deficits may directly contribute to tau pathology and disease progression. This is further supported by *in vitro* experiments which showed that Nup98 triggers tau aggregation and accelerates tau fibrilization seen in Alzheimer's disease and tauopathy brains.[60]

Despite these functional insights into tau phenotypes and nucleocytoplasmic transport deficits, it remains to be established whether the karyopherin abnormalities observed in Alzheimer's disease patient brain[55-59,107] are a cause or a consequence of tau pathology. A possible pathogenic mechanism might be related to the functional link between nuclear pore proteins enriched in FG-motifs like Nup98 and karyopherins of the beta family. These FG-motif Nups and KPNBs are known to interact with each other at the nuclear membrane[110,111] and their functional interactions are essential to allow repeated Ran-dependent cycles of nucleocytoplasmic shuttling.[112] These data suggest that alterations in levels or localisation of FG-Nups, Ran and/or KPNBs could trigger tau pathology - and vice versa – and lead to the cellular and molecular phenotypes already observed in transgenic mouse models of FTD-Tau.[60]

### Karyopherin abnormalities in synucleinopathies

Synucleinopathies are a group of neurodegenerative disorders that include Parkinson's disease (PD), Parkinson's disease with dementia (PDD), dementia with Lewy bodies (DLB) and multiple system atrophy (MSA).[113,114] Their common pathological feature is the accumulation and aggregation of α-synuclein (α-syn) in Lewy bodies (LB) found in neurons and glial cells, and in Lewy neurites (LN) within neuronal processes.[66,115-117] Native α-syn is found in presynaptic terminals of neurons where it acts as a chaperone for neurotransmitter release.[118] Its initial discovery in *Torpedo* identified α-syn not only in synaptic terminals but also in neuronal nuclei,[119] suggesting a physiological function in both synapse and the nucleus. Indeed, recent experiments using transgenic mice and human cell culture revealed translocation of α-syn to the nucleus where it modulates DNA repair,[120] raising the question how α-syn can shuttle into the nucleus.

α-syn does not possess a nuclear localisation signal and due to its small size of 14kDa[121] may enter the nucleus via passive diffusion. Recent findings however, identified three intermediates that interact with α-syn and together translocate into the nucleus: by direct binding of α-syn to the retinoic acid receptor and its calcium-dependent nuclear import;[122] by sumoylation of α-syn and its subsequent binding to KPNA6 which mediates transport into the nucleus;[123] by interaction with TRIM28[124] and its karyopherin-alpha mediated nuclear import.[125] Dysfunction of either pathway has been directly implicated in α-syn pathology in transgenic mice and in cell culture experiments expressing human mutant forms of α-syn, as well as in post-mortem brain tissue of DLB cases.[120,122,126] These findings could explain the pathological phenotypes observed in synucleinopathies in which α-syn has been detected in aberrant forms in the nucleus[120,127,128] and in presynaptic terminals[129-132] where it can spread across synaptically connected brain regions.[133,134]

Consistent with these findings, karyopherin abnormalities have been associated with synucleinopathies. Alterations in KPNB1 expression were identified using microarray analysis of tissue material derived from PD patients with a triplication in the α-syn encoding *SNCA* gene.[65,135] Lewy body formation triggered by prefibrillary α-syn was found to alter the expression levels of KPNA7 and XPO1/exportin-1.[66] The PD-related α-syn mutation G51D was shown to cause nuclear aggregation,[136] while nuclear aggregation of α-syn was shown to be mitigated by targeted knockdown of KPNA2.[62] Cell culture studies revealed that α-syn mediated cytotoxicity caused upregulation of KPNB3[61] and involved interaction with KPNA3.[137] In addition to these phenotypes directly related to α-syn accumulation, several other studies reported karyopherin abnormalities in cell and animal models of familial forms of PD that are characterised by α-syn pathology. KPNA2 and KPNA6 have been identified as substrates of LRRK2/PARK8[64] whose mutations cause one of the most common forms of PD.[138,139] Mutant DJ-1/PARK7[140] was shown to interact with KPNB2 in an oxidative stress-dependent manner leading to its mislocalisation.[63] FBXO7/PARK15 which requires XPO1 for its normal localisation[141] was found mislocalised together with α-syn in Lewy bodies, Lewy neurites and cytoplasmic inclusions in glial cells in both PD and MSA cases.[142]

Despite this multitude of phenotypes, it should be noted that unlike in ALS, FTD and AD cases, conspicuous karyopherin pathology has not been reported in any of the synucleinopathies to date. It therefore seems likely that the detected phenotypes described above are a consequence rather than a cause contributing to the cellular and molecular mechanisms underlying synucleinopathies. However, this situation may change with future work specifically focusing on karyopherin abnormalities and their potential role in the onset and progression of α-syn pathology.

### Karyopherin abnormalities in cell and animal models of Huntington's disease

Huntington's disease is an autosomal dominant disorder characterised by neurodegeneration and gliosis in the striatum and cortex,[143] leading to progressive loss of motor function, psychiatric symptoms, and cognitive impairment.[144] HD is caused by a CAG triplet repeat expansion encoding for a polyglutamine (poly-Q) tract in the N-terminus of Huntingtin (HTT).[145] Expansions of >36–39 Glutamine residues trigger disease, and longer poly-Q stretches above this threshold cause early onset Huntington's disease.[146,147] Huntingtin is a large (~350 kDa) protein that shuttles between the cytoplasm and nucleus;[148] it contains a XPO1/CRM1-dependent nuclear export signal at its carboxyl-terminus[149,150] and its nuclear import occurs via its PY-NLS by the TNPO/KPNB2 pathway.[68,151] In mutant Huntingtin, the poly-Q domain is flanked by the first 17 amino acids at the N-terminus (Nt17) and by a poly-Proline stretch at its C-terminus. Mutant Huntingtin is more prone to proteolysis, resulting in smaller fragments of the N-terminus containing the poly-Q expansion of exon 1.[152] Nt17 plays an important role in Huntingtin aggregation and toxicity, in addition to acting as another nuclear export signal via interactions with XPO1/CRM1 in an α-helical and RanGTP-dependent manner.[149]

The subcellular localisation and actual function of wildtype Huntingtin is still debated,[153,154] however there is consensus that poly-Q expanded mutant Huntingtin causes gain of function proteinopathy in both the nucleus and the cytoplasm.[144] Consistent with this hypothesis, both intranuclear and cytoplasmic aggregates have been observed in HD patient brains[155,156] while cell culture experiments using human embryonic kidney 293T cells, neuroblastoma cells and mouse primary neurons transfected with fragments of mutant Huntingtin revealed its nuclear and cytoplasmic aggregation.[70] Moreover, in the striatum and cortex of HD mouse models, mutant Huntingtin formed both nuclear and cytoplasmic aggregates, which resulted in the accumulation of mRNA in the nucleus and an overall reduction in mRNA levels.[70,157] These cellular and molecular phenotypes may point towards a role of nucleocytoplasmic transport deficits in HD.

Indeed, poly-Q and mutant Huntingtin pathology has been related to nuclear pore complex deficits and defective nucleocytoplasmic transport, including karyopherin abnormalities.[67,69,150,158-161] Transfection of mouse primary neurons with mutant Huntingtin expanded with 96 poly-Q fragments not only caused nuclear and cytoplasmic aggregation of mutant Huntingtin; it also caused aggregation of KPNA2 and KPNA4.[70] Some of these cytoplasmic KPNA2/4 aggregates were found associated with mutant Huntingtin aggregates, suggesting that Huntington's disease-related poly-Q pathology directly causes karyopherin abnormalities. Consistent with this hypothesis, affinity-purification mass spectrometry using mouse brain tissue expressing mutant Huntingtin expanded with 97 poly-Q, identified the karyopherins KPNA6, XPO7, CAS and KPNB1 as binding partners of mutant Huntingtin.[158] Several of these karyopherins were independently confirmed as targets of mutant Huntingtin in a genomics and proteomics study using transgenic mice expressing human mutant Huntingtin exon 1 carrying disease-related CAG repeats.[159] These experiments identified altered expression levels of the karyopherin alpha proteins KPNA1, KPNA2 and KPNA3 together with KPNA4 and KPNA6, as well as altered expression levels of KPNB1 and KPNB2.[159] Despite these experimental evidences in cell and animal models of Huntington's disease, conspicuous karyopherin pathology has not been reported so far in brain of patient cases.

Interestingly, however, it has been shown that HD-related CAG repeat expansion RNAs can also function as a template for repeat-associated non-AT (RAN) translation. The resulting homo-polymeric RAN translation products include poly-Ala, poly-Ser, poly-Leu, and poly-Cys peptides that have been detected in brains of Huntington's disease patients, especially in regions of microglial activation and degenerative apoptotic cell loss, as well as throughout the degenerating cerebellar layers of juvenile-onset Huntington's disease cases.[162] These data indicate that the aetiology of Huntington's disease may share similarities with C9ALS/FTD, both in its RAN translation-derived peptide accumulation[161,163] and in nuclear transport deficits.[70,161] However, further work is required to establish whether karyopherin pathology characterises patient brain cases and whether these phenotypes are mere consequences of, or directly contribute to mutant Huntingtin, poly-Q and RAN-derived peptide pathology that have been detected in cell and animal models of Huntington's disease.

## Phase transition and protein aggregation of karyopherin cargos

Despite their functional heterogeneity and disease-specificity, TDP-43, tau, α-syn and FUS as well as mutant Huntingtin with poly-Q, all harbour low complexity regions rendering them intrinsically disordered proteins.[9,164-167] Low complexity regions are devoid of charged amino acids but contain polar ones (Q, N, S, G) with interspersed aromatic residues (F, Y), making these chains highly flexible and interactive.[168] Because of their similarity to infectious yeast proteins, low complexity regions are also called prion-like domains.[5] Recent functional studies provide compelling evidence that prion-like low complexity regions make intrinsically disordered proteins vulnerable to changes in the surrounding milieu which can alter their physical properties, eventually leading to their accumulation in pathological aggregates.[13] These changes in aggregate state are similar to phase separations of mixed solutions, ranging from liquid-liquid de-mixing into liquid droplets, to phase transitions into gel-like structures (hydrogels) that over time can mature into solid fibrillary aggregates such as amyloid-like fibres.[169,170] These phase transitions, especially the initiating phase separation of soluble proteins with low complexity regions into droplets and hydrogels, tend to accumulate in membrane-less compartments such as P granules, paraspeckles, nucleoli and stress granules and are very sensitive to changes in temperature, salt, pH or molecular concentration.[169,170] These environmental alterations characterize protein accumulation and cellular stress observed in neurodegenerative diseases.[12-14]

Recent studies showed that tau,[166,171-174] mutant Huntingtin-poly-Q,[165] TDP-43,[9,175-178] FUS[7,17-19,179,180] and α-syn[167,181] can undergo liquid-liquid phase separation. In the case of poly-Q pathology, it was shown that Huntingtin exon 1 aggregates are present in liquid-like as well as solid-like forms.[165] The poly-Q and proline-rich regions of exon 1 facilitate liquid-like formation, which can convert into solid-like assemblies when poly-Q length expands beyond the threshold for Huntington's disease.[165] Similar to poly-Q related phase separation, recent studies showed that phosphorylated tau, recombinant mutant aggregate prone tau, and soluble phospho-tau isolated from human AD brain can all undergo phase separation.[166,171-174] These findings are consistent with previous studies, in which TIA1, a major component of stress granules, promoted tau misfolding and aggregation by sequestering tau into stress granules, whilst TIA1 knockout inhibited tau mediated pathology and toxicity.[182]

### Karyopherin-mediated phase transition in neurodegeneration

Previously known for their role in nucleocytoplasmic transport, karyopherins are now also recognised for their important functions as chaperones and molecular switches in the pathogenesis of neurodegenerative proteinopathies **(Figure 3)**.[13,14,183] In the case of TDP-43 and FUS, recent studies established that this process is mediated by karyopherins acting as molecular chaperones in phase separation.[17-20] These data suggest that karyopherin abnormalities are not only a common denominator in neurodegenerative proteinopathies but misregulation of karyopherins can also trigger onset and progression of disease. In line with this notion, *in vitro* studies demonstrated that TDP-43 which assembled into protein-rich droplets via liquid-liquid phase separation could fibrillate over time.[8,94] Liquid-liquid phase separation of TDP-43 is crucially dependent on the C-terminal low complexity region,[175] especially its alpha-helical structure[9,178,184] and three tryptophan residues within it, W334, W385 and W412.[185] Using hydrogels, Guo and colleagues[19] showed that KPNA and KPNB1 interact with the canonical nuclear localisation signal of TDP-43 **(Figure 3)**, the binding of which reverses and prevents its aberrant phase transition.[19,102] ALS-related mutations in the low complexity region of TDP-43 accelerate the transition of liquid-like droplets to pathologically fibrillized states,[8,186] whereas overexpression of KPNA and KPNB1 resulted in reversal and prevention of TDP-43 fibrillization.[19] Moreover, studies in sporadic and familial forms of ALS and FTD revealed that disease-initiating cytoplasmic accumulation of TDP-43 is associated with a vicious cycle of KPNA dysfunction that is accompanied by depletion of KPNB1 and a dysfunctional nuclear pore complex.[15,16,20,93]

Comparable phenotypes have been observed in FUS-related ALS. TNPO1/KPNB2 was shown to inhibit liquid-liquid phase separation of FUS[18] and to reverse and prevent the fibrillization of FUS and other RNA binding proteins by interacting with the PY-NLS domain.[19] Using NMR spectroscopy and small angle X-ray scattering techniques, Yoshizawa *et al.*[18] showed that a combination of strong and weak binding between TNPO1/KPNB2 and FUS inhibits phase separation. The interactions between TNPO1/KPNB2 and FUS competed with FUS-FUS

interactions, thereby disrupting phase separation.[18] Moreover, *in vitro* studies revealed that TNPO1/KPNB2 suppresses FUS phase separation and stress granule association by interacting with its arginine residues in its PY-NLS and RGG domains.[17] These studies also showed that ALS-related mutations in FUS reduce its sensitivity to the chaperone activity of TNPO1/KPNB2 both in cells and *in vitro*, thereby leading to a model in which FUS mutations both enhance phase separation and elude the chaperone activity of TNPO1/KPNB2.[17-19] Arginine residues appear to be critical to aberrant phase separation and karyopherin-mediated chaperoning as overexpression of arginine-containing DPRs derived from RAN translated, C9ALS/FTD related G4C2 repeat RNA, can induce the assembly of stress granules, triggering the co-localisation of Ran and karyopherins into these membraneless organelles.[187] The resulting decrease in karyopherin availability impairs karyopherin-mediated cargo transport and chaperone function, thereby increasing cytoplasmic accumulation and phase transitions, which ultimately leads to disease-related fibrillization and aggregate formation.

Corresponding molecular and cellular phenotypes have been observed in AD, FTD-Tau and synucleinopathies. Liquid-liquid phase separation of tau protein was shown to initiate tau aggregation[166,174,188,189] that was significantly enhanced by FTD-tau associated mutations including P301L-tau linked to inherited tauopathy.[173] Tau positive neurofibrillary tangles and Hirano bodies were found enriched in KPNA2/Importin-α1 aggregates,[56,57] while KPNB1 and XPO5 were found co-localised with hyperphosphorylated tau in affected neurons.[59] α-syn has been shown to undergo spontaneous liquid-liquid phase separation which was accelerated by disease-related mutations A53T, E46K and S129E and formation of fibrils[167] that could mature into Lewy body-like assemblies.[181] Of note, Lewy body formation related to synucleinopathies has been associated with altered KPNA7 and XPO1/exportin-1 expression,[66] while inhibition of KPNA2 impaired the pathological accumulation of α-syn in the nucleus.[62] Similarly, triggered by its disease-related poly-Q tracts, mutant huntingtin exon1 was shown to form liquid-like assemblies that could convert to solid fibrillar structures.[165]

These findings suggest that karyopherin abnormalities are interlinked with alterations of aggregation-prone proteins that can phase separate into disease-related intracellular inclusions and aggregates. In line with these observations, work on nucleocytoplasmic transport receptors, including KPNB3, showed that karyopherins can act as molecular chaperones, shielding basic domains of positively charged proteins and preventing their aggregation.[102,187] Together, these data indicate that both karyopherin abnormalities and affected karyopherin-cargo binding promote phase transition which accelerates protein mislocalisation and aggregate formation characteristic of neurodegenerative proteinopathies. As a result of these new findings, it is becoming increasingly evident that karyopherins can act as chaperones comparable to molecular disaggregases[14,190,191] that protect aggregation-prone proteins against misfolding, accumulation and irreversible phase-transition into insoluble aggregates. It is therefore reasonable to consider karyopherins as therapeutic targets for the treatment of neurodegenerative diseases for which currently no cure or effective treatments are available.

## Targeting karyopherin abnormalities in degenerative proteinopathies

Current treatments like Riluzole for ALS[192] or Donepezil, Rivastigmine, Galantamine and Memantine for AD[193] offer only short-term amelioration of clinical symptoms but do not arrest progressive cell loss. Similarly, PD-related treatment with Levodopa offers symptomatic improvements but it does not arrest synapse loss and subsequent neurodegeneration.[118] However, there is time between when a patient is first diagnosed and when severe mental and physical decline occurs, indicating the existence of a potential therapeutic window during which surviving, unaffected neurons could be protected against their progressive dysfunction leading to further cognitive decline and degenerative cell loss. As biomarkers for early diagnosis and effective treatments become available, the development of therapies to halt progressive cell death becomes an increasingly hopeful prospect. Karyopherins have already been described as diagnostic and prognostic biomarkers in cancer and viral infections, where they are usually upregulated.[194-196] As a result, several compounds have been developed to target nuclear import and export receptors[197] and phase III clinical trials are under way targeting the KPNA/KPNB1 interface highjacked by viruses causing Dengue fever and COVID-19, respectively.[196]

In neurodegenerative diseases, karyopherins are generally downregulated and mislocalized, which together with impaired nucleocytoplasmic transport and defective chaperone function leads to aberrant phase transition and the subsequent accumulation and aggregation of affected proteins.[14,191] Recent studies demonstrate that targeting karyopherins can reverse aberrant phase transition and extract aggregated proteins from stress granules.[17-20,102] Thus, targeting karyopherins, especially their chaperone function, provides a potential novel therapeutic avenue to shield and maintain vulnerable proteins in their native and soluble state, thereby protecting them against phase transition, mislocalisation and aberrant aggregate formation, ultimately protecting affected neurons against cytotoxicity and degenerative loss.

The role of compounds inhibiting nuclear transport of specific cargo substrates has been reported for their use as anti-cancer and anti-viral agents, with more than 20 nuclear transport inhibitors being identified,[196-198] including Mifepristone, Ivermectin and Verdinexor.[199-202] Inhibitors of XPO1/CRM1 like Verdinexor, commonly also known as selective inhibitors of nuclear export (SINE) compounds, have been developed via structure-based drug design.[198] Previous studies investigating SINE compounds focused on their use as anti-cancer and anti-viral agents,[197] however they are also candidates for the development of therapeutic strategies to target neurodegeneration.[203] For example, XPO1 inhibition in preclinical models of inflammatory demyelination and axonal damage resulted in attenuation of disease progression.[203] Comparably, in ALS models, XPO1 inhibition showed neuroprotective effects against C9orf72-related disease phenotypes caused by G4C2 hexanucleotide repeat expansion.[21] Targeted manipulation of the nuclear import receptor karyopherin-β2 was able to reverse the defects caused by mutant FUS.[19,204] Pharmacological treatment with Verdinexor and KPT-276, two XPO1 inhibitor, reduced cell death by more than 50% caused by selective overexpression of C-terminal fragments of human TDP-43 or its ALS-related mutant Q331K in mouse cortical neurons.[15] A parallel study showed that SINE compounds targeting XPO1 could prolong, at least to some extent, neuronal survival of rodent primary neurons and mitigate motor symptoms in an *in vivo* rat model expressing wildtype and mutant forms of TDP-43.[205] This study revealed that none of the SINE compounds could enhance nuclear TDP-43 levels, while depletion of XPO1 or other exportins had little effect on TDP-43 localization,[205] consistent with recent studies suggesting that nuclear egress of TDP-43 does not require XPO1-mediated nuclear export.[52,53]

These findings suggest that pharmacological targeting of karyopherin function might represent a new strategy for disease-modifying treatments of neurodegenerative proteinopathies. However, given the different topology of disease-related protein accumulation – nuclear and/or cytoplasmic - a more balanced application will be required that considers not only nuclear transport inhibitors but also their targeted gain of function which will require careful assessment and calibration. Karyopherins modulate an essential cellular process, energy-dependent nucleocytoplasmic transport of cargo proteins required for cellular homeostasis.

Any interference or manipulation of this process may therefore cause unwanted side effects affecting the localisation and function of cargo proteins essential for neuronal and glial function. It will be of great interest to await and analyse the outcome of currently ongoing clinical trials targeting the KPNA/KPNB1 interface as a strategy to combat several types of cancer such as leukemia and glioblastoma, as well as viral infections including Dengue fever and COVID-19.[196,197,206] If proven to be clinically safe, some of these compounds might be suitable for repurposing as a novel therapeutic strategy for the targeted treatment of neurodegenerative proteinopathies.

## Conclusions and future directions

Nucleocytoplasmic transport is a fundamental process that moves macromolecules across the nuclear membrane, thereby allocating proteins to their principal destination of activity, which is an essential requirement for proper cell function and viability. Key players in this process are karyopherins that mediate the transport of cargo proteins in and out of the nucleus. The mislocalisation of proteins and their subsequent accumulation in the nucleus or cytoplasm are characteristic features, and likely the direct cause, of several neurodegenerative proteinopathies. Why these proteins mislocalise and accumulate in the first place, has remained elusive and the lack of insights into the underlying pathogenic mechanisms constitutes a major obstacle for the development of efficient therapeutic strategies to go beyond current symptomatic treatments.

Despite their differences in function, ranging from nucleic acid processing by TDP-43, to tau-mediated microtubule stabilisation and chaperone activity of alpha-synuclein in synaptic terminals, all these proteins harbour low complexity regions that make them intrinsically vulnerable to phase transitions of their aggregation state. Phase transitions can be initiated by changes in cellular acidity, temperature and alterations in pH or salt but also by protein mutations, all of which have been associated with neurodegeneration. However, *in vivo* determination of these pathomechanisms, their detection in patient tissue and a direct role in disease formation and/or progression remain to be shown, and although first generation preclinical biomarkers of liquid-liquid phase separation are becoming available, their use and efficiency remains to be established.[207]

Nucleocytoplasmic transport deficits and in particular karyopherin abnormalities are now recognised as a major player, and possibly a trigger, in mediating the mislocalisation and accumulation of disease-related proteins in degenerative proteinopathies, especially in ALS and FTD. Furthermore, karyopherins can also act as chaperones by shielding proteins from irreversible phase transitions, thereby retaining their targets in a soluble form and thus devoid of disease-related aggregation comparable to disaggregase activity. It is currently unclear and remains to be elucidated, how these chaperone functions are exerted, whether they include specific amino acids/protein interaction domains and how these interactions are accomplished. Thus, an exciting area of future research will be to determine how karyopherins exert their chaperone and disaggregase activity. Work in this area will lead to both a better understanding of how neurodegenerative diseases develop and may uncover novel treatment opportunities.

Pharmacological targeting of karyopherins represents a promising new strategy for therapeutic intervention in neurodegenerative diseases. It remains a major task for an ever-growing elderly population to identify therapies that can stop or at least ameliorate degenerative cell loss. Even after decades of intense research, the destiny of a patient remains the same: when they enter the hospital with disease-specific clinical symptoms, a significant number of neurons are already lost, while remaining functional ones are doomed to degenerate over time due to the lack of efficient treatments. Several compounds targeting karyopherins are already in clinical trials to treat specific forms of cancer and viral infections, with an emphasis on the karyopherin-α/karyopherin-β1 interface. A number of neurodegenerative diseases are characterised by intracellular proteinaceous inclusions and aggregates for which nucleocytoplasmic transport-modifying compounds may yield promising outcomes for an effective treatment of these devastating illnesses. Targeting nuclear transport may therefore be an advantageous strategy for halting and perhaps reversing the progression of neurodegenerative proteinopathies.

## Acknowledgements

We thank D. Solomon, R.B. Parsons and M. Atwal for comments on the manuscript.

## Funding

This work was supported by a Bolashak International scholarship (Sharipov/Aug28/2019) of the government of Kazakhstan to D.S.; Slovenian Research Agency grants (P4-0127, J3-9263,

J3-8201, J7-9399 and N3-0141) and CRP-ICGEB research grant (CRP/SVN19-03) to B.R.; the Hungarian Brain Research Program (2017-1.2.1-NKP-2017-00002) and NKFIH-SNN-132999 grant to T.H.; and an Alzheimer's Research UK - King's College London Network grant to F.H. and T.H. F.H. acknowledges funding from the UK Medical Research Council (G0701498; MR/L010666/1); the UK Biotechnology and Biological Sciences Research Council (BB/N001230/1); Alzheimer's Research UK (Hirth/ARUK/2012); and the UK MND Association (Hirth/Oct07/6233; Hirth/Mar12/6085; Hirth/Oct13/6202; Hirth/Nov15/914-793; Hirth/Oct16/890-792).

## Competing interests

The authors declare no competing interests.

**Figure 1. Functional architecture of karyopherins. (A)** Karyopherin alpha (KPNA) family proteins consist of two main functional domains: an importin beta (KPNB) binding (IBB) domain for nuclear import and export and Armadillo repeats for the recognition of canonical nuclear localisation signal (cNLS) of cargo proteins. The IBB consists of cNLS binding boxes (bars in purple), major ('KRR') and minor ('RRRR' or 'RRQR' or 'RRHR') binding sites, which in the absence of KPNB occupy the cNLS-binding surface of Armadillo repeats. This prevents import of 'unloaded' karyopherin complexes in the nucleus. KPNA family proteins are subdivided into three subfamilies, alpha 1, 2 and 3, based on differences in amino acid sequence in their IBB domain and cNLS binding sites. See Table 1 for details on all KPNA family members. **(B)** Karyopherin beta (KPNB) family proteins comprise an N-terminal (20-120 a/a) importin domain responsible for binding with Ran and for directed protein translocation across the nuclear envelope, and HEAT repeats that are distinguishable by their flexibility and recognition/binding of cargo-proteins via the NLS or the nuclear export signal and binding to KPNA. Shown are two functionally important representatives of the KPNB family, KPNB1 and CAS; see Table 2 for details on all KPNB family members. Protein sequences were obtained from UniProt (see Table 1 and Table 2 for ID numbers); Clustal Omega was used for sequence alignment. *Abbreviations*: KPNA, karyopherin alpha; KPNB, karyopherin beta/; cNLS, canonical nuclear localisation signal; NES, nuclear export signal; CAS, cellular apoptosis susceptibility protein.

**Figure 2: Nucleocytoplasmic transport and chaperone function of karyopherins. Left**, schematic diagram showing the RanGTP/GDP cycle through the nuclear pore complex. In the cytoplasm, RanGAP1, together with RanBP, hydrolyses RanGTP to maintain high cytoplasmic concentrations of RanGDP. In the nucleus, RCC1 (nuclear RanGEF) facilitates GTP-GDP exchange, causing high concentrations of RanGTP. These regulators maintain higher concentrations of RanGDP in the cytoplasm whilst preserving high levels of RanGTP in the karyoplasm, leading to a gradient required for energy-dependent nucleocytoplasmic transport. **Right**, schematic diagram of the classical nuclear import and export pathway. During nuclear import, KPNB1 binds to KPNA, which itself is bound to the classical nuclear localisation signal (cNLS) of cargo, forming a trimeric complex. KPNA also exerts chaperone function to cargo-cNLS by shielding basic residues from hydrophobic/ionic interactions, which maintains a cargo protein in its native soluble state. KPNB1 carries the complex through the nuclear pore complex, where RanGTP binds, causing a conformational change in the bound importin (note, some KPNBs shown as beta bind directly to cargo forming a dimeric complex which directly translocates to the nucleus). This results in a trimeric complex of KPNA, nuclear export factor CAS, and RanGTP, and a dimeric complex consisting of KPNB1 and RanGTP. Both complexes then translocate back to the cytoplasm where their respective RanGTPs are hydrolysed to bind to the next cargo. During export, Exportin/KPNB (beta) bound to RanGTP binds to the cargo-NES of the cargo in the nucleoplasm. This complex is exported through the nuclear pore complex into the cytoplasm where RanGTP is hydrolysed, which triggers cargo release. *Abbreviations*: KPNA, karyopherin alpha; KPNB, karyopherin beta; cNLS, canonical nuclear localisation signal; CAS, cellular apoptosis susceptibility protein; RAN, Ras-related nuclear; RCC1, regulator of chromosome condensation 1; Ran-GAP, Ran GTPase activating protein; RanBP1, Ran binding protein 1; RanGEF, Ran Guanine Exchange Factor.

**Figure 3. Karyopherins abnormalities in neurodegenerative proteinopathies. Left:** In ALS/FTD-TDP, cytoplasmic accumulation of TDP-43 and its aggregates not associated with stress granules have been shown to sequester KPNAs. **Right**: In AD and FTD-Tau, pathological tau has been shown to sequester KPNAs. See text and Table 3 for details. *Abbreviations*: ALS, amyotrophic lateral sclerosis; FTD-TDP, frontotemporal dementia with TDP-43 pathology; Ad, Alzheimer's disease; FTD-Tau, frontotemporal dementia with Tau pathology; KPNA, karyopherin alpha; KPNB1, karyopherin beta 1; CAS, cellular apoptosis susceptibility.

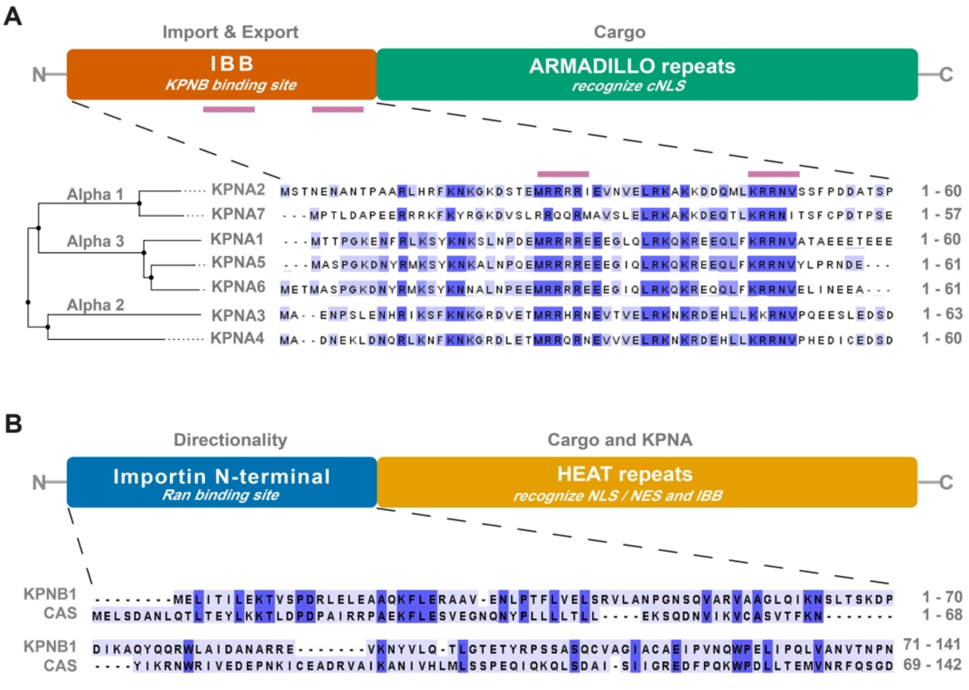

Pasha et al Figure 1

181x125mm (300 x 300 DPI)

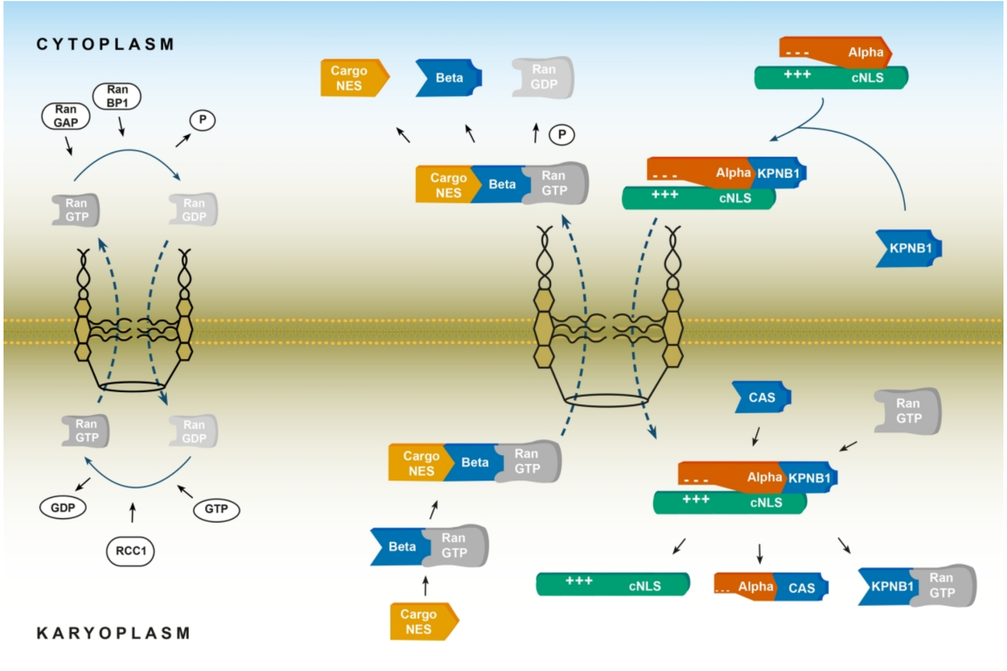

Pasha et al Figure 2

173x116mm (300 x 300 DPI)

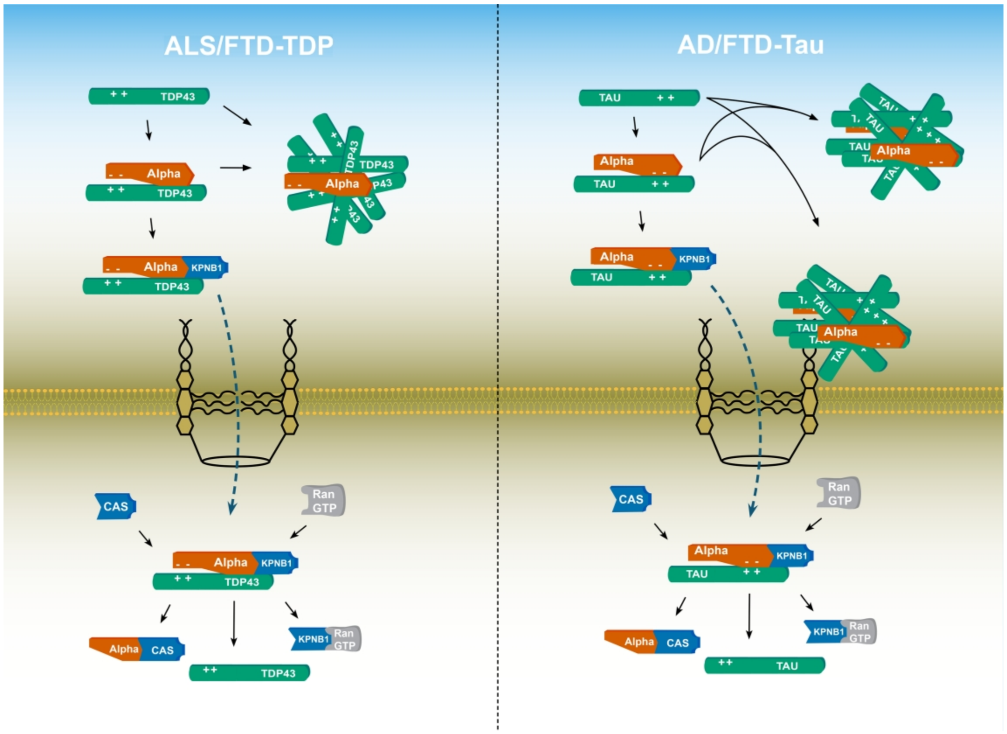

Pasha et al Figure 3

269x196mm (300 x 300 DPI)

Table 1 Karyopherin Alpha proteins and their essential cargos

| Gene/ Protein Name | NCBI ID | Sub Family | Synonyms | | | | Cargos | References |
|---|---|---|---|---|---|---|---|---|
| | | | Importin | SRP | NPI/QIP | Other | | |
| KPNA1 | 3836 | α3 | IPOA-5 | SRP1β | NPI-1 | RCH2 | TDP-43 NF-κB (p50/p65; c-Rel; p52; RelB) | Pumroy *et al.*,[27] Lee *et al.*,[50] Nishimura *et al.*[54] |
| KPNA2 | 3838 | α1 | IPOA-1 | SRP1α | NPI-3 | RCH1 | TDP-43 NF-κB (p65) | Pumroy *et al.*,[27] Nishimura *et al.*[54] |
| KPNA3 | 3839 | α2 | IPOA-4 | SRP4 hSRP1 SRP1γ | QIP2 | n/a | TDP-43[a] NF-κB (p50/p65; p52) ataxin-3 α1ACT | Pumroy *et al.*,[27] Nishimura *et al.*,[54] Khsoravi *et al.*,[208] Sowa *et al.*,[209] Tsou *et al.*,[210] Fagerlund *et al.*[211] |
| KPNA4 | 3840 | α2 | IPOA-3 | SRP3 | QIP1 | MGC12217 | TDP-43[a] NF-κB (p50/p65; p52) | Pumroy *et al.*,[27] Nishimura *et al.*[54] 208, 211 |
| KPNA5 | 3841 | α3 | IPOA-6 | SRP6 | n/a | n/a | TDP-43 NF-κB (p50/p65; c-Rel; p52; RelB) | Pumroy *et al.*,[27] Nishimura *et al.*[54] |
| KPNA6 | 23633 | α3 | IPOA-7 | n/a | NPI-2 | MGC17918 | TDP-43 NF-κB (c-Rel; RelB) | Pumroy *et al.*,[27] 54 |
| KPNA7 | 402569 | α1 | IPOA-8 | n/a | n/a | n/a | n/a | n/a |

*Abbreviations*: IPOA, importin alpha; KPNA, Karyopherin alpha; MGC, Mammalian Gene Collection;NF-κB, nuclear factor-κB; NPI, Nucleoprotein interactor; QIP, *Importin alpha Q;* RCH, RAG cohort protein; SRP, signal recognition particle; TDP-43, transactive response (TAR) DNA binding protein of 43 kDa.

[a]Whilst TDP-43 acts as a cargo for KPNA1-6, KPNA3 & 4 show the strongest interactions.

Table 2 Karyopherin Beta proteins and their essential cargos

| Gene/Protein | | | | Cargos | References |
|---|---|---|---|---|---|
| Name | NCBI ID | Synonyms | Full name | | |
| XPO1 | 7514 | EXP1 | Exportin-1 | TDP-43?; rRNA; snRNA; mRNA | Archbold *et al.*[205] Lu *et al.*[212] |
| | | CRM1 | Chromosomal Maintenance 1 | | |
| CAS | 1434 | XPO2 | Exportin-2 | Importin α; TDP-43? | Kutay *et al.*[37] Nishimura *et al.*[54] |
| | | CAS | Cellular apoptosis susceptibility | | |
| | | CSE1L | Chromosome segregation 1-like | | |
| XPOT | 11260 | XPO3 | Exportin-3 | tRNA; Scyl1 | Kutay *et al.*,[38] Chafe *et al.*[213] |
| XPO5 | 57510 | EXP5 | Exportin-5 | Scyl1miRNA precursors | Chafe *et al.*[213] Wang *et al.*[214] |
| | | RANBP21 | Ran-binding protein 21 | | |
| XPO6 | 23214 | EXP6 | Exportin-6 | Actin | Stueven *et al.*[215] |
| | | RANBP20 | Ran-binding protein 20 | | |
| XPO7 | 23039 | EXP7 | Exportin-7 | p50RhoGAP; TDP-43; Tubulin; Histones | Archbold *et al.*,[205] Mingot *et al.*,[216] Aksu *et al.*[217] |
| | | RANBP16 | Ran-binding protein 16 | | |
| KPNB1 | 3837 | IPO1 | Importin-1 | HTT; RPL23A; RPS7; RPL5; SNAI1 PRKCI | Desmond *et al.*[68] Jaekel and Görlich[218] UniProtKB-Q14974 |
| | | IPO90 | Importin-90 | | |
| | | IPOB | Importin-beta | | |
| | | IMB1 | Importin beta 1 subunit | | |
| | | KPNB1 | Karyopherin-beta 1 | | |
| | | PTAC97 | Pore targeting complex 97 kDa subunit | | |
| | | NTF97 | Nuclear factor p97 | | |
| TNPO1 | 3842 | TRN | Transportin 1 | HTT; FUS; TAF15; EWS; hnRNP-a1/a2; RPL23A; RPS7; RPL5; ADAR/ADAR1 (isoform 1 and 5) | Desmond *et al.*,[68] Jaekel and Görlich,[218] Neumann *et al.*,[219] Barraud *et al.*[220] |
| | | IPO2 | Importin beta-2 | | |
| | | KPNB2 | Karyopherin-beta 2 | | |
| | | MIP | M9 region interaction protein (MIP) | | |
| TNPO2 | 30000 | TRN2 | Transportin-2 | FUS; hnRNP A1/H/M | Güttinger *et al.*[221] |
| | | IPO3 | Importin 3 | | |
| | | KPNB2B | Karyopherin β2-2B | | |
| TNPO3 | 23534 | TRN3 | Transportin-3 | SFRS1; SFRS2 | Kataoka *et al.*[222] |
| | | TRNSR | Transportin-SR | | |
| | | IPO12 | Importin-12 | | |
| | | LGMD1F | Limb girdle muscular dystrophy 1F | | |
| IPO4 | 79711 | IMP4 | Importin-4 | RPS3A | Jaekel *et al.*[223] |
| | | IMP4b | Importin-4b | | |
| | | RANBP4 | Ran-binding protein 4 | | |
| IPO5 | 3843 | IMP5 | Importin-5 | RPL23A RPS7; RPL5 | Jaekel and Görlich[218] |
| | | IMB3 | Importin subunit β-3 | | |
| | | KPNB3 | Karyopherin β-3 | | |
| | | RANBP5 | Ran-binding protein 5 | | |
| | | PSE1 | Protein Secretion Enhancer 1 | | |
| IPO7 | 10527 | IMP7 | Importin-7 | RPL23A RPS7; RPL5 | Jaekel and Görlich[218] |
| | | RANBP7 | Ran-binding protein 7 | | |
| IPO8 | 10526 | IMP8 | Importin-8 | SRP19 | Dean *et al.*[224] |
| | | RANBP8 | Ran-binding protein 8 | | |
| IPO9 | 55705 | IMP9 | Importin-9 | Actin; RPS7 RPL18A RPL6 | Jaekel *et al.*[223] UniProtKB - Q96P70 |
| | | RANBP9 | Ran-binding protein 9 | | |
| | | KIAA1192 | Identified by Kazusa Institute | | |
| | | HSPC273 | Homo sapiens HSPC273 mRNA, partial cds | | |
| IPO11 | 51194 | IMP11 | Importin-11 | UbcM2 | Plafker and Macara[225] |
| | | RANBP11 | Ran-binding protein 11 | | |
| XPO4 | 64328 | EXP4 | Exportin-4 | n/a | n/a |
| IPO13 | 9670 | IMP13 | Importin-13 | RBM8A SUMO1; UBC9; EIF1A | Mingot *et al.*[226] |
| | | KAP13 | Karyopherin 13 | | |
| | | RANBP13 | Ran binding protein 13 | | |
| | | KIAA0724 | Identified by Kazusa Institute | | |
| | | LGL2 | Late gestation lung 2 | | |

Table 3 Karyopherin abnormalities in neurodegenerative proteinopathies

| Karyopherin | Pathogenesis | References |
|---|---|---|
| ALS/FTD-TDP | | |
| KPNA2 | Reduced levels in FTD-TDP frontal cortex;<br>Accumulating TDP-43 causes cytoplasmic mislocalization in *Drosophila* model of C9ALS/FTD. | Chou *et al.*,[15]<br>Solomon *et al.*,[16]<br>Nishimura *et al.*[54] |
| KPNA4 | Accumulating TDP-43 causes cytoplasmic mislocalization in *Drosophila* model of C9ALS/FTD;<br>Depletion of KPNA directly contributes to impaired nuclear import and cytoplasmic accumulation of TDP-43. | Chou *et al.*,[15]<br>Solomon *et al.*,[16]<br>Park *et al.*[103] |
| XPO1 | In ALS models, XPO1 inhibition showed neuroprotective effects against c9orf72 related disease. | Zhang *et al.*[21] |
| CAS | Reduced levels in FTD-TDP frontal cortex;<br>Knockdown of CAS dysregulates the import of TDP-43. | Nishimura *et al.*[54] |
| KPNB1 | Reduced levels in spinal cords of patients with ALS;<br>Irregular and disrupted nuclear staining in sporadic ALS with TDP-43;<br>Depletion of KPNB1 directly contributes to impaired nuclear import and cytoplasmic accumulation of TDP-43;<br>ALS related mutations in FUS reduce its sensitivity to the chaperone activity of KPNB1, ultimately leading to increased phase separation. | Solomon *et al.*,[16]<br>Hofweber *et al.*,[17]<br>Yamashita *et al.*,[78]<br>Aizawa *et al.*,[80]<br>Park *et al.*[103] |
| TNPO 1 | ALS related mutations in FUS reduce its sensitivity to the chaperone activity of TNPO1, ultimately leading to increased phase separation. | Hofweber *et al.*[17] |
| AD/FTD-Tau | | |
| KPNA2 | Accumulation and aggregation of KPNA2 is found in neurofibrillary tangles and Hirano bodies of hippocampal CA1 neurons of AD patients. | Lee *et al.*,[56]<br>Carter[57] |
| KPNA3 | Abnormally upregulated levels of KPNA3 found in cDNA microarray studies of AD human brains. | Wang *et al.*[55] |
| KPNA6 | Upregulated KPNA6 identified in association with small non-coding RNAs. | Roy *et al.*[58] |
| KPNB1 | Found within cytoplasmic granules in hippocampal neurons in AD cases and co-localizes with hyperphosphorylated tau. | Nuovo *et al.*,[59]<br>Sheffield and Mirra[107] |
| KPNB2 | Found in cytoplasmic granules in hippocampal neurons and in tangle-bearing cells of AD cases. | Sheffield and Mirra[107] |
| XPO1 | Tau-induced nuclear envelope invaginations sequester XPO1 in a Drosophila model of tauopathy. | Cornelison *et al.*[108] |
| Synucleinopathies | | |
| KPNA2 | Targeted knockdown linked to nuclear aggregation of $\alpha$-syn;<br>Substrate of LRRK2/PARK8. | Ma *et al.*,[62]<br>Han *et al.*[64] |
| KPNA3 | $\alpha$-syn mediated cytotoxicity involves interaction with KPNA3 | Büttner *et al.*[137] |
| KPNA6 | Substrate of LRRK2/PARK8 | Han *et al.*[64] |
| KPNA7 | Lewy body formation triggers alterations in the expression level of KPNA7. | Ma *et al.*[62] |
| XPO1 | FBXO7/PARK15 was found mislocalised together with $\alpha$-syn in Lewy bodies, Lewy neurites and cytoplasmic inclusions in glial cells in both PD and MSA cases;<br>Lewy body formation triggers the alterations in the expression level. | Ma *et al.*,[62]<br>Zhao *et al.*[142] |
| KPNB1 | Alterations in the expression level of PD patients with a triplication in the SNCA locus encoding $\alpha$-syn. | George *et al.*,[65]<br>Devine *et al.*[135] |
| KPNB2 | Mutant DJ-1/PARK7 was shown to interact with KPNB2 in an oxidative stress-dependent manner leading to its mislocalisation. | Björkblom *et al.*[63] |
| KPNB3 | $\alpha$-syn mediated cytotoxicity lead to upregulation of KPNB3. | Zhou *et al.*[61] |
| KPNA1 | Target of mHtt in genomics and proteomics study of transgenic mice expressing mHtt. | Langfelder *et al.*[159] |
| Huntington's disease | | |
| KPNA2 | mHtt-transfected mouse neurons cause aggregation of mHtt and KPNA2/KPNA4. Some KPNA2/4 aggregates were associated with mHtt aggregates;<br>Target of mHtt in genomics and proteomics study of transgenic mice expressing mHtt. | Woerner *et al.*[70],<br>Langfelder *et al.*[159] |
| KPNA3 | Target of mHtt in genomics and proteomics study of transgenic mice expressing mHtt. | Langfelder *et al.*[159] |
| KPNA4 | mHtt-transfected mouse neurons cause aggregation of mHtt and KPNA2/KPNA4. Some KPNA2/4 aggregates were associated with mHtt aggregates;<br>Target of mHtt in genomics and proteomics study of transgenic mice expressing mHtt. | Woerner *et al.*,[70]<br>Langfelder *et al.*[159] |
| KPNA6 | Binding partner of mHtt in mouse brain expressing mHtt expanded with 97 poly-Q;<br>Target of mHtt in genomics and proteomics study of transgenic mice expressing mHtt. | Shirasaki *et al.*,[158]<br>Langfelder *et al.*[159] |
| KPNB1 | Binding partner of mHtt in mouse brain expressing mHtt expanded with 97 poly-Q;<br>Target of mHtt in genomics and proteomics study of transgenic mice expressing mHtt. | Shirasaki *et al.*,[158]<br>Langfelder *et al.*[159] |
| KPNB2 | Target of mHtt in genomics and proteomics study of transgenic mice expressing mHtt. | Langfelder *et al.*[159] |
| XPO7 | Binding partner of mHtt in mouse brain expressing mHtt expanded with 97 poly-Q. | Shirasaki *et al.*[158] |
| CAS | Binding partner of mHtt in mouse brain expressing mHtt expanded with 97 poly-Q. | Shirasaki *et al.*[158] |

*Abbreviations*: $\alpha$-syn, alpha-synuclein; AD, Alzheimer's disease; ALS, amyotrophic lateral sclerosis; FTD-TDP, frontotemporal dementia with TDP-43 pathology; FTD-Tau, frontotemporal dementia with Tau pathology; KPNA, karyopherin alpha; KPNB, karyopherin beta; MSA, multiple system atrophy; PD, Parkinson's disease.
